# The Analysis of Teachers’ Perceptions of Moral Education Curriculum

**DOI:** 10.3389/fpsyg.2022.967927

**Published:** 2022-07-26

**Authors:** Quankun Zhang, Norzihani Binti Saharuddin, Nor Azni Binti Abdul Aziz

**Affiliations:** Faculty of Educational Studies, Universiti Putra Malaysia, Selangor, Malaysia

**Keywords:** teachers’ perceptions, moral education curriculum, ATLAS.ti 9, thematic review, framework

## Abstract

With the development of teachers’ psychological cognition, the moral education curriculum develops as per the changes in times. Currently, a resurgence of interest on studying teachers’ perceptions of the moral education curriculum has observed. This is because moral education curriculum of each country plays a unique role in the overall design of the country’s education curriculum. However, studies on teachers’ perceptions of the moral education curriculum are scarce, and no framework has been developed to guide the teaching and learning of teachers for the moral education curriculum for schools. The moral education curriculum serves as one of the main vehicles for value education, with teachers as direct executors. It can influence the overall development of the school system and long-term development of a country. Thus, in this paper, we reviewed current literature related to teachers’ perceptions of the moral education curriculum and developed future trends considering teachers’ perceptions of the moral education curriculum in improving existing moral education programs in a country. We identified a list of keywords related to moral education curriculum and teacher perceptions and relevant to the scope of this study. These keywords were used to search the articles from the Scopus and WoS studies. Overall, 32 papers meeting the search criteria were retrieved. Further, we conducted thematic studies, and four themes were successfully identified: the model of teacher perceptions of moral and national education, the dimensions of the teacher ethics model (framework), teaching strategies, and the role of teachers. The results of this study suggested a new framework for research trends in teachers’ perception models, particularly toward the moral education curriculum. This study contributed to the knowledge regarding teaching and learning approaches of moral education considering teachers’ perceptions of the moral education curriculum. The findings of study would benefit future studies and would improve the implementation of a country’s moral education program.

## Introduction

Teachers’ perceptions of the curriculum have received increasing attention from researchers, giving rise to a fever of teachers’ perceptions. Several authors have described teachers’ perceptions of their experiences of teaching values infused into the curriculum and the impact of these lessons on their students (Kumarassamy and Koh, 2019). Cognition and perception belong to the realm of psychology. Kagan (1990) proposed that teacher cognition is defined as pre-service or in-service teacher self-reflection; beliefs and knowledge about teaching, students, and content; and awareness of problem-solving strategies specific to classroom instruction. Borg (2003) noted that teacher cognition is initially shaped by teachers’ school education and professional experience and refers to cognitive structures such as knowledge, beliefs, and thoughts. Therefore, this paper adopts teachers’ perception of moral education curriculum.

Asif et al. (2020) suggested that teachers’ perceptions and beliefs about the goals of moral education and areas students must learn are essential because teachers play a critical role in the moral development of their students. A proposal was made to use a model of teachers’ perceptions of moral and national education to investigate the factors influencing teachers’ perceptions of the curriculum (Wong et al., 2021). The direction of research on teachers’ perceptions of the curriculum is related to the teaching of English (Soleimani and Lovat, 2019; Chen and Abdullah, 2022) and science (Kumarassamy and Koh, 2019). Moreover, recent trends in research focus on teachers’ perceptions of the moral curriculum as a new theme for future research.

Cheung and Wong (2002) argued that different curricular value orientations reflect teachers’ different perspectives on the purpose of education, importance of education, and teacher–student interactions. Thus, curricular value orientations are an expression of curricular perspectives. Yurdakul (2015) noted that teachers’ concept of curriculum and the formation of teachers’ concept of education is a rich, holistic understanding of objects, people, and thing and their unique relationships with each other. Teachers’ concept of curriculum, as a type of teachers’ educational concept, will be changed by the changing practical experience of teachers. Cronin-Jones (1991) and Cheung and Ng (2000) noted that teachers’ perspective on curriculum, teachers’ conception of Johnston (1990) proposed that teachers’ image on curriculum can be seen as the teacher’s perception of the curriculum. Teachers’ image of the curriculum is a personal, meta-level, and organized form of knowledge that teachers develop in their practice. It is the experience teachers develop in the course and gain new experiences in new techniques. At the same time, he emphasized different understandings of the curriculum by teachers. For example teachers express similar conceptions of the curriculum. Still, the images associated with their verbal expressions of the pictures may differ considerably; therefore, the teaching strategies they choose and actions they take will be different as well.

As far as the development of teachers’ perception of moral education curriculum is concerned, teachers’ perceptions of the moral education curriculum can be traced back to Beddoe (1981). Subsequent research by several scholars has extended the understanding of teachers’ perceptions of the moral curriculum. Welsh et al. (2019) examined how teachers’ perceptions and practices have shifted in response to the threat of state takeover and explored how the accountability pressures associated with the threat of state takeover have led to organizational change at the school level. On the other hand, research on teachers’ perceptions of the moral curriculum can contribute to education at schools, the development of students, and policymakers. Moreover, it can fill gaps and contribute to the construction of curricula that can make curricular goals a reality in the classroom (Roux and Dasoo, 2020). For students, it can bring changes in behaviors and greater interest in participating in society (Luguetti and McDonald, 2020). It can improve existing teacher education, promote teachers’ professional development (Thornberg et al., 2013), promote patriotic education, and give students a global perspective (Wong et al., 2017; O’Flaherty and McCormack, 2019).

However, despite the growing body of research on teachers’ perceptions of the moral education curriculum, no thematic review papers are available regarding this. No framework has been developed through literature review to guide instruction process for teachers for the moral education curriculum. Therefore, this paper focused on selected literature that discussed teachers’ perceptions of the moral education curriculum and answered the following questions.

**Research Question 1.** What are current trends in teachers’ perceptions of Moral Education Curriculum?
**Research Question 2.** What is the framework of teachers’ perceptions of Moral Education Curriculum?

## Methodology

This study used the thematic review approach with ATLAS.ti 9. the thematic review is a tool introduced by Zairul (2020, 2021), which was used because this research method applies the thematic analysis procedure in the literature review process. Clarke and Braun (2013) defined thematic analysis as the identification of the model and construction of themes during an in-depth reading of the topic. In this study, the following steps were taken to identify patterns and build themes for teachers’ perceptions and trends in the moral education curriculum. The study’s goal was to analyze and interpret the findings to suggest future research on teachers’ perceptions of the moral curriculum. The literature was selected according to several criteria: at least the keyword(s) teachers’ perception, teachers’ perspective, teachers’ views, and morals.

The sources for the literature were the Web Science databases of Clarivate Analytics and Scopus of Elsevier. The Science Network was selected for all journals indexed with an impact factor measured in the Journal Citation Report (Jamal et al., 2008). Scopus was selected because it has the largest collection of peer-reviewed literature. A filter was applied in the Web of Science using “type of documents,” article types, and proceedings papers, but not reviews. The remaining data sets contained the analysis criteria: “Title, Keywords, and Abstract.” The following keywords were used as search terms: teachers’ perception, teachers’ perspective, teachers’ views, moral education, and moral education curriculum (Table 1).

**Table 1:** Search strings.

SCOPUS	(TITLE-ABS-KEY (“teachers’ perceptions”) AND TITLE-ABS-KEY (moral AND education AND curriculum))	15 results
	(TITLE-ABS-KEY (“teachers’ views”) AND TITLE-ABS-KEY (moral AND education AND curriculum)) AND (EXCLUDE (DOCTYPE, “ch”) OR EXCLUDE (DOCTYPE,"re”))	5 results
	(TITLE-ABS-KEY (“teachers’ perspectives”) AND TITLE-ABS-KEY (moral AND education AND curriculum))	1 results
Web of Science	“teachers’ perceptions” (All Fields) and moral education curriculum (All Fields) and 4TH WORLD CONFERENCE ON EDUCATIONAL SCIENCES WCES (Exclude—Conference Titles) and Early Access (Exclude—Document Types)	13 results
	“teachers’ perceptions” (All Fields) and “moral education” (All Fields) and FUTURE ACADEMY MULTIDISCIPLINARY CONFERENCE ICEEPSY CPSYC ICPSIRS BE CI (Exclude—Conference Titles)	14 results
	“teachers’ views” (All Fields) and “moral education” (All Fields) and Proceedings Papers (Exclude—Document Types) and Turkish (Exclude—Languages)	4 results

**Figure 1:**
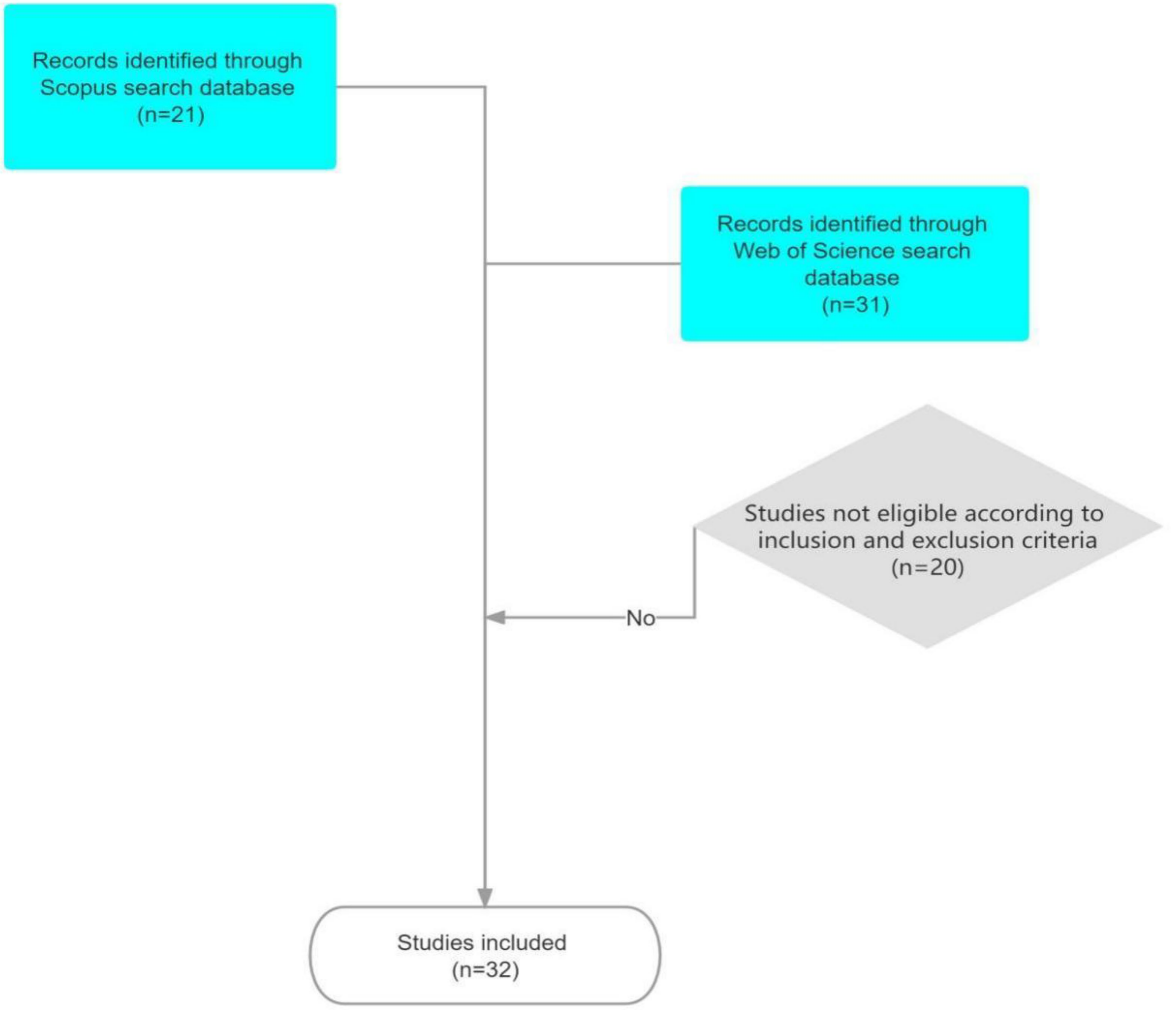
Inclusion and exclusion criteria from Zairul (2021) for thematic review (TR). Reproduced with permission.

**Figure 2:**
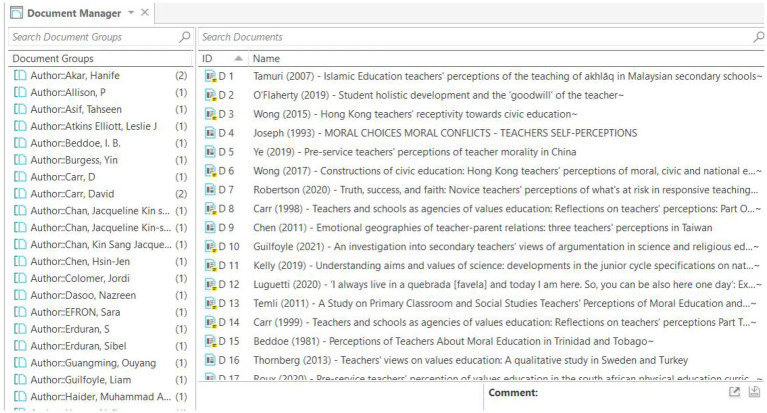
Metadata generated in ATLAS.ti Scientific Software Development GmbH [ATLAS.ti 9 Windows] (2022). We used ATLAS.ti Windows (Version 9.1.3.0) to complete our work. Reproduced with permission.

The articles were uploaded to the Mendeley site for data processing. Data processing involved deleting duplicate reports, updating authors’ names, and verifying the accuracy of metadata (Figures 1, 2). From Mendeley, the 32 pieces were exported to ATLAS.ti 9 for analysis of current trends in the literature. Multiple bibliometric data were created from the bibliographic list, including the title of the article, year, country of the author, journal, keywords used, and field. The resulting articles are subdivided into quantitative and qualitative sections. The quantitative section reported data obtained from a numerical perspective, whereas the qualitative section identified themes from selected articles and developed a conceptual framework.

## Results

**Figure 3:**
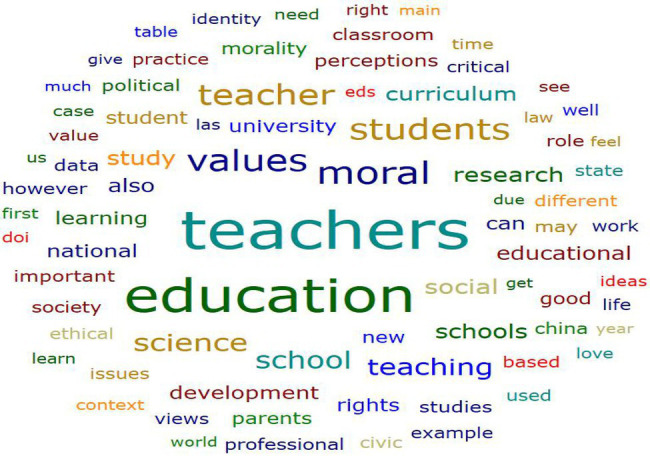
Word cloud on word frequencies from 32 retrieved documents.

The results are divided into two parts: quantitative and qualitative. The results of the quantitative part are based on the analysis of 32 documents in the main document, generating the word cloud below (Figure 3). The most powerful words appearing in the cloud, for example, “moral education,” “education,” “teachers,” “curriculum,” and “perception” indicated a high frequency in the articles. As mentioned above, the focus of this article is on teachers’ perceptions and moral education curriculum.

There are no review articles on teachers’ perceptions and frameworks to build curricula for moral education despite an increasing trend. A research protocol was developed, and the data were collected, prepared, and interpreted based on previous research and in a logical order. From the analysis of the word “cloud,” the word “teacher” was mentioned 3,702 times, followed by “education” (3,134 times), and moral and perceptions, perceptions were mentioned 1721, 394, and 262 times, respectively. The number of relevant publications has increased over the years. After 2017, 1, 3, 5, and 3 articles were published in 2018, 2019, 2020, and 2022 (at the time of writing), respectively. We would like to note that this analysis does not appear to be excluded from any limitations or comprehensive analysis, as it focuses on search strings, indexing, and exclusion criteria. However, we state that it reflects the literature based on research questions.

### Quantitative Results

From our analysis below, several journals were selected by us related to teachers’ perceptions. According to this list, Journal of moral education, Teaching and Teacher Education, and Journal of Beliefs and Values were the three most popular choices. If the keywords used in this search were only “teacher perceptions,” the number of articles found would be more than 8,000. However, the number significantly declined after focusing the search string on moral education courses. They were more focused, thus proving that the topic is still new and could be explored more in the future. However, the growing interest suggests that the use of terms “moral education curriculum” and “teachers’ perceptions” has increased. This can be seen in the evolutionary development of the articles given in Table 2. Moreover, in terms of geographic dissemination, research related to teachers’ perceptions of the moral curriculum is trending in the United States, United Kingdom, and Hong Kong, China.

**Table 2:** No. of articles according to the periodical.

	1981	1993	1998	1999	2003	2007	2008	2011	2012	2013	2014	2015	2017	2018	2019	2020	2021	Totals
Asia Pacific Educ	-	-	-	-	-	-	-	1	-	-	-	-	-	-	-	-	-	1
Citizenship Teaching and Learning	-	-	-	-	-	-	-	-	-	-	-	1	-	-	-	-	-	1
Citizenship, Social and Economic Education	-	-	-	-	-	-	-	-	-	1	-	-	-	-	-	-	-	1
Compare	-	-	-	-	-	-	-	-	-	-	-	-	1	-	-	-	-	1
Educational Research	-	-	-	-	-	-	-	-	-	-	-	-	-	-	1	-	-	1
Educational Sciences: Theory and Practice	-	-	-	-	-	-	-	1	-	-	-	-	-	-	-	-	-	1
European Physical Education Review	-	-	-	-	-	-	-	-	-	-	-	-	-	-	-	1	-	1
International Journal of Educational Research	-	-	-	-	-	-	-	-	-	1	-	-	-	-	-	-	-	1
International Studies in Sociology of Education	-	-	-	-	-	-	-	-	-	-	1	-	-	-	-	-	-	1
Irish Educational Studies	-	-	-	-	-	-	-	-	-	-	-	-	-	-	1	-	-	1
Journal of Beliefs and Values: Studies in Religion and Education	-	-	1	1	-	-	-	-	-	-	-	-	-	-	-	-	1	3
Journal of Educational Change	-	-	-	-	-	-	-	-	-	-	-	-	-	-	-	-	1	1
Journal of moral education	1	1	-	1	1	1	-	-	-	-	-	-	2	-	-	-	-	7
Journal of Teacher Education	-	-	-	-	-	-	-	-	-	-	-	-	-	-	-	-	1	1
Peace and Conflict Studies	-	-	-	-	-	-	-	-	-	-	-	-	-	-	-	1	-	1
Res Sci Educ	-	-	-	-	-	-	-	-	-	-	-	-	-	-	1	-	-	1
Science Teacher Education	-	-	-	-	-	-	-	-	-	-	-	-	-	-	-	1	-	1
Social Responsibility Journal	-	-	-	-	-	-	-	-	-	-	-	-	-	1	-	-	-	1
South African Journal of Childhood Education	-	-	-	-	-	-	-	-	-	-	-	-	-	-	-	1	-	1
Sustainability	-	-	-	-	-	-	-	-	-	-	-	-	-	-	-	1	-	1
Teaching and Teacher Education	-	-	-	-	-	-	1	1	-	-	-	-	-	-	1	-	-	3
The Curriculum Journal	-	-	-	-	-	-	-	-	1	-	-	-	-	-	-	-	-	1
Totals	1	1	1	2	1	1	1	3	1	2	1	1	3	1	4	5	3	32

In the United States, this can be seen in the paper by Joseph and Efron (1993), who discussed teachers’ self-perceptions in moral choices and moral conflicts. Rique and Lins-Dyer (2003) explored teachers’ views on forgiveness for conflict resolution among students in schools, whereas LePage et al. (2011) compared Turkish and American teachers’ views on morality and moral education. Johnson et al. (2017) presented a student assessment in a model of ethical dilemma decision-making, and Robertson and Atkins Elliott (2020) focused on novice teachers’ perceptions of the risks faced by science-responsive teaching. In the United Kingdom, Carr and Landon (1998) studied research on reflections on teachers’ perceptions. Allison et al. (2012) studied perceptions of interdisciplinary learning as an ethical enterprise. Hung (2013) explored teachers’ perceptions of liberal and communitarian constructs. Guilfoyle et al. (2021) studied secondary school teachers’ perceptions of science and religious education arguments. In Hong Kong, China, Wong et al. (2021) examined teachers’ perceptions of civic education, whereas Zhao (2014) explored teachers’ perceptions of their relationship with the state in China. Interestingly, in recent years, attention has been paid to teachers’ perceptions of moral education in mainland China; for example, Ye and Law (2019) focused on pre-service teachers’ perceptions of teacher ethics. Li and Tan (2017) explored Chinese teachers’ perceptions of “good citizenship.” Asif et al. (2020) discussed ethics education for sustainable development and university teachers’ perceptions of moral education.

Several studies from Turkey studied teachers’ perceptions of moral education and its development and learning in elementary classrooms and social studies (Temli et al., 2011). The story of teacher education by comparing teachers’ perceptions of moral education in the United States and Sweden was reported by LePage et al. (2011) and Thornberg et al. (2013), respectively. Two articles were from Taiwan and China; Chen and Wang (2011) explored the perceptions of the relationship between teachers and parents from teachers’ perspectives.

Although the literature is sourced from 32 articles, they were from 37 countries. Among them, the duplicated countries were United States, Turkey, Hong Kong, China, Taiwan, and United Kingdom. Regarding the indicated regional dispersion (Table 3), research on teachers’ perceptions of the curriculum has been conducted in many countries for quite some time, for example, in the United States, United Kingdom, Australia, Ireland, Sweden, Finland, Turkey, Malaysia, and Jamaica. More recently, in Asia, research on teachers’ perceptions of moral education has begun in Hong Kong, China. Research on teachers’ perceptions of moral education curricula has already emerged in mainland China, but more research is needed. Some authors mentioned the dimensions of the teacher spirituality model (Johnson et al., 2017; Ye and Law, 2019). Some authors have studied the teachers’ moral and national education perception model and further developed the model by continuously proposing new factors to build the model (Wong et al., 2015, 2017, 2021). Some authors emphasized the role of teaching strategies and reported the process to integrate teaching of moral education with their own experiences, which is the direction of some authors’ research and shows teachers’ perceptions of moral education curriculum (Allison et al., 2012; Kelly and Erduran, 2019; Guilfoyle et al., 2021). Some authors emphasized the teacher’s role in the perception of moral education (Carr and Landon, 1998, 1999; Ye and Law, 2019). It is important to note that the trends in Europe and the United States are mainly in the application. In Asia, especially in China and Hong Kong, China, the research revolves around the policies and frameworks in which they operate.

**Table 3:** The distribution of articles according to country.

	1981	1993	1998	1999	2003	2007	2008	2011	2012	2013	2014	2015	2017	2018	2019	2020	2021	Totals
Australia	-	-	-	-	-	-	-	-	-	-	-	-	-	-	-	1	-	1
China	-	-	-	-	-	-	-	-	-	-	-	-	1	-	1	1	-	3
Colombia	-	-	-	-	-	-	-	-	-	-	-	-	-	-	-	1	-	1
Finland	-	-	-	1	-	-	-	-	-	-	-	-	-	-	-	-	-	1
Hong Kong, China	-	-	-	-	-	-	-	-	-	-	1	1	1	-	-	-	1	4
Ireland	-	-	-	-	-	-	-	-	-	-	-	-	-	-	2	-	-	2
Israel	-	-	-	-	-	-	-	-	-	-	-	-	-	-	-	-	1	1
Jamaica	-	-	-	-	-	-	-	-	-	-	-	-	-	1	-	-	-	1
Malaysia	-	-	-	-	-	1	-	-	-	-	-	-	-	-	-	-	-	1
Pakistan	-	-	-	-	-	-	-	-	-	-	-	-	-	-	-	1	-	1
Singapore	-	-	-	-	-	-	-	-	-	-	-	-	-	-	1	-	-	1
South African	-	-	-	-	-	-	-	-	-	-	-	-	-	-	-	1	-	1
Sweden	-	-	-	-	-	-	1	-	-	1	-	-	-	-	-	-	-	2
Taiwan, China	-	-	-	-	-	-	-	1	-	1	-	-	-	-	-	-	-	2
Tobago	1	-	-	-	-	-	-	-	-	-	-	-	-	-	-	-	-	1
Trinidad	1	-	-	-	-	-	-	-	-	-	-	-	-	-	-	-	-	1
Turkey	-	-	-	-	-	-	-	2	-	1	-	-	-	-	-	-	-	3
United Kingdom	-	-	1	1	-	-	-	-	1	1	-	-	-	-	-	-	1	5
United States	-	1	-	-	1	-	-	1	-	-	-	-	1	-	-	1	-	5
Totals	2	1	1	2	1	1	1	4	1	4	1	1	3	1	4	6	3	37

In Table 4, the trends and patterns of the publications selected in this study are described. After merging and renaming the initial codes, the codes yielded four themes. The following themes are discussed in detail in the qualitative section.

**Table 4:** Authors according to themes.

	Model of teachers’ perceptions of moral and national education	Framework	Teaching strategy	The role of the teacher
Tamuri, 2007			√	
Allison et al., 2012			√	
Asif et al., 2020			√	√
Roofe, 2018			√	
Chen and Wang, 2011			√	
Carr and Landon, 1998				√
Carr and Landon, 1999				√
Guilfoyle et al., 2021			√	
Hung, 2013			√	
Beddoe, 1981				√
O’Flaherty and McCormack, 2019				√
Johnson et al., 2017		√		
Rique and Lins-Dyer, 2003		√		
Tirri, 1999		√		
Kumarassamy and Koh, 2019			√	
LePage et al., 2011			√	
Li and Tan, 2017			√	
Luguetti and McDonald, 2020			√	
Joseph and Efron, 1993				
Perry-Hazan and Neuhof, 2021				
Pineda and Meier, 2020			√	
Kelly and Erduran, 2019			√	
Robertson and Atkins Elliott, 2020				
Roux and Dasoo, 2020			√	√
Temli et al., 2011				√
Thornberg, 2008			√	
Thornberg et al., 2013			√	
Wong et al., 2015	√			
Wong et al., 2017	√			
Wong et al., 2021	√			
Ye and Law, 2019		√	√	√
Zhao, 2014	√			

### Qualitative Results

**Figure 4:**
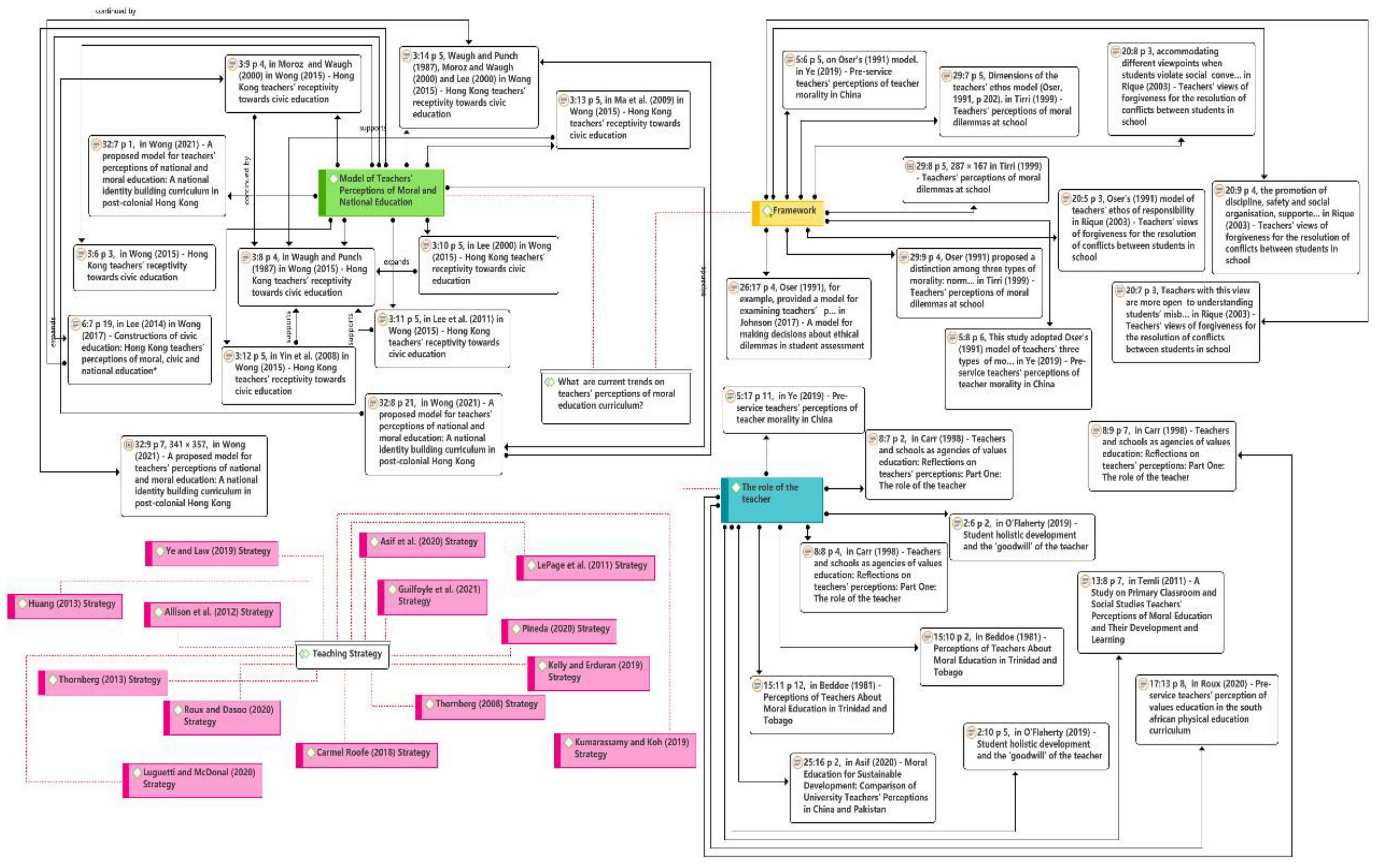
The overall thematic review formulation.

The qualitative section focuses on the themes identified in response to the research questions. Four themes have been defined based on the article’s focus and topic. The following themes were identified from the selected publications: model of teachers’ perceptions of moral and national education, dimensions of the teachers’ ethos model, teaching strategy, and the role of the teacher. The main themes are not independent but intersect across the articles presented in this paper. Some articles tend to use multiple themes and vice versa. In the next section (Model of Teachers’ Perceptions of Moral and National Education, Framework, Teaching Strategy, and The Role of the Teacher), the themes are discussed separately and in more detail to answer research question 1 (What are the current trends in teachers’ perceptions of moral education curriculum?). The next section (A Proposed Conceptual Framework for Teachers’ Perceptions of Moral Education Curriculum) describes the formulation of the framework (Figure 4).

What are the current trends in teachers’ perceptions of moral education curriculum?

#### Model of Teachers’ Perceptions of Moral and National Education

**Figure 5:**
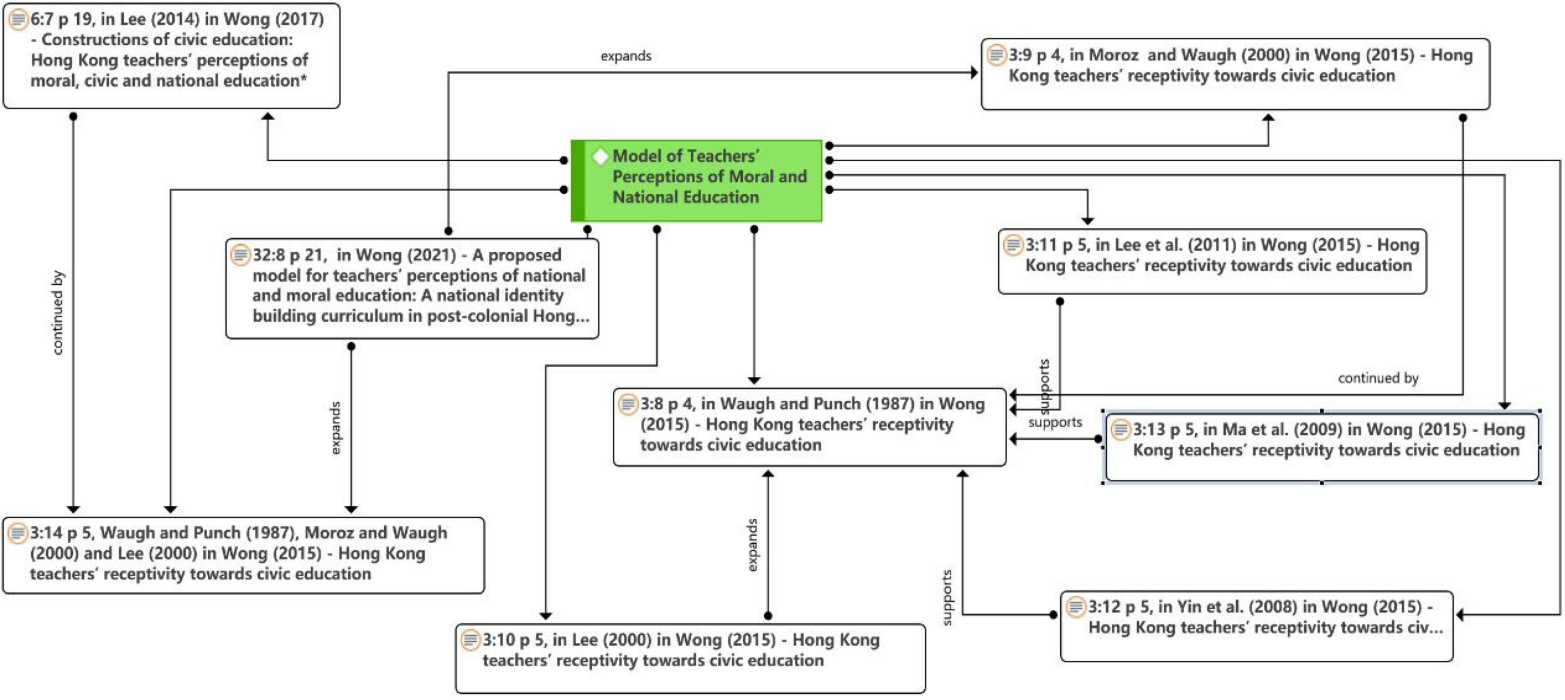
Network on the model of teachers’ perceptions of moral and national education theme.

As seen in Figure 5, in this mode, the variable of teachers’ acceptance of the system-wide change (hereafter referred to as the teacher acceptance model) was proposed by Waugh and Punch (1987). Building on the work of Waugh and Punch (1987), Chi-Kin Lee (2000), and Moroz and Waugh (2000), teachers’ perceptions of the moral education model were constructed.

Chi-Kin Lee (2000) used a teacher receptivity model with additional variables or factors, including seven variables (attitude toward the guidelines, behavioral intention, perceived non-monetary cost-effectiveness, perceived usefulness of the policies, perceived school support, perceived support from others, and concerns) to examine teachers’ receptivity to environmental education curriculum reform in Hong Kong.

Further, the teacher receptivity model was modified and applied to the study of curriculum innovation. To examine Australian high school teachers’ receptivity to educational change, Moroz and Waugh (2000) used the “teacher receptivity model,” which included overall feelings, attitudes, behavioral intentions, and behaviors as dependent variables.

Furthermore, Yin et al. (2008) used structural equation modeling to construct the model’s validity, providing a new way of thinking to develop and validate a model of teachers’ perceptions of moral education. Some authors later studied teachers’ perceptions of the new curriculum reformation in mainland China. Ma et al. (2009) investigated teachers’ acceptance of mainland China’s high school curriculum reformation. Lee and Yin (2011) used the teacher acceptance model to study teachers’ approval of the curriculum reformation in mainland China. Lee (2014) applied the teachers’ perception model to study civic education in Singapore.

Finally, in recent years, Wong et al. (2015, 2017, 2021) focused on the study of teachers’ acceptance of civic education in Hong Kong, China, and developed teachers’ perceptions of the moral education model by adding Hong Kong characteristics to the original teachers’ perceptions of the model. Wong et al. (2021) suggested that socio-political and media factors should be added to the model of teachers’ perceptions to explain the factors that influence teachers’ acceptance of curriculum reformation.

It can be seen that Waugh and Punch (1987) proposed the model; Waugh and Punch (1987), Chi-Kin Lee (2000), and Moroz and Waugh (2000) extended the model; Chi-Kin Lee (2000) and Moroz and Waugh (2000) developed the model; Yin et al. (2008), Ma et al. (2009), Lee and Yin (2011), and Lee (2014) supported the development of the model; and Wong et al. (2015, 2017, 2021) extended the development of the model.

Current research lacks more methods to examine the model of teachers’ perceptions of the moral curriculum, and the variables need to be updated. The model of teachers’ perceptions of the moral curriculum changes with times. It is crucial to consider the circumstances of each region in the study, and only then the model of teachers’ perceptions of moral education curriculum will be refined and developed and can serve moral education and teacher education better. Further, the model of teachers’ perceptions of moral education curriculum can be combined with other theories to open new dimensions to the study of teachers’ perceptions of moral education curriculum.

#### Framework

**Figure 6:**
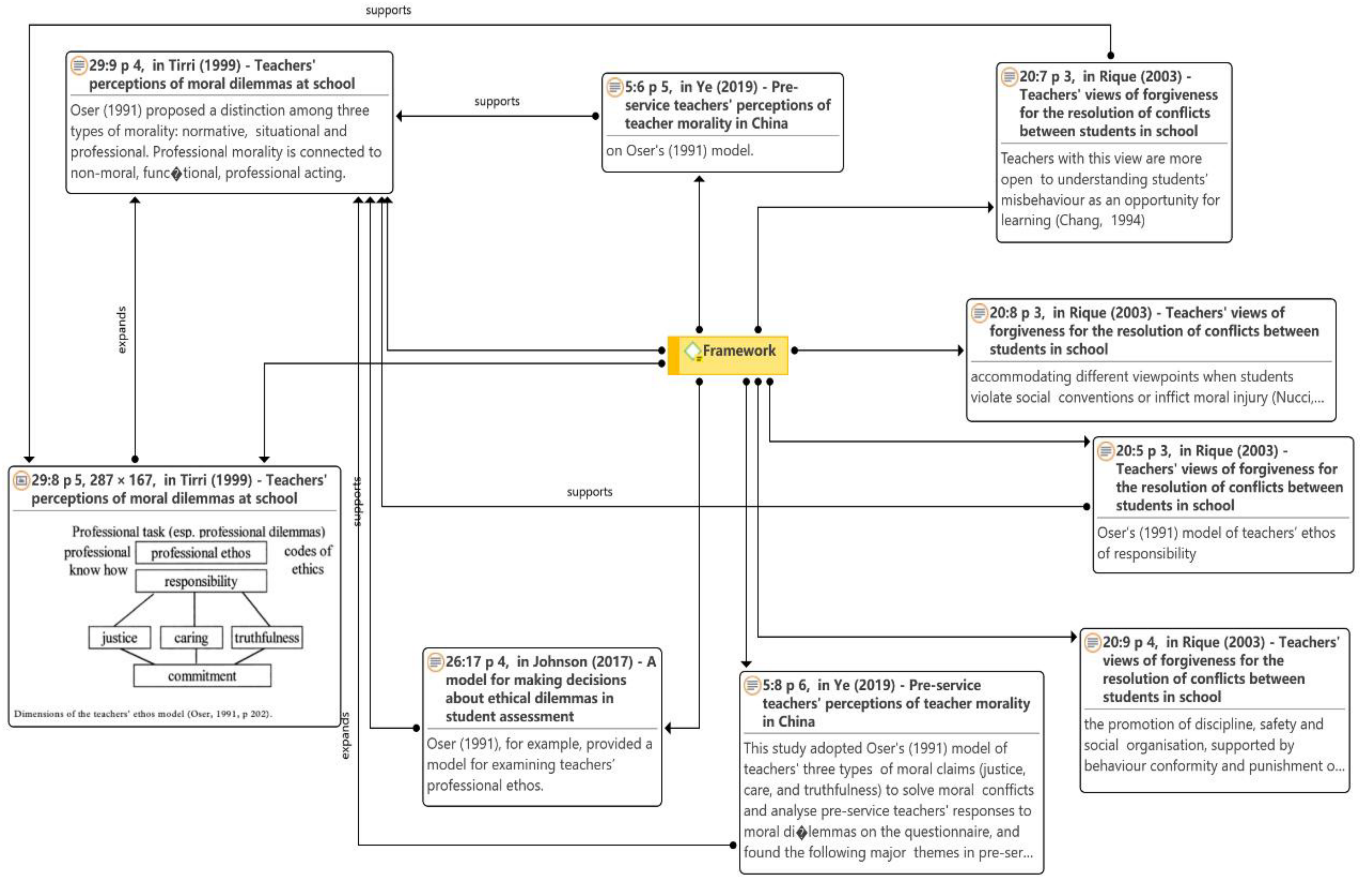
Network on the framework theme.

As seen in Figure 6, the dimensions of the teacher ethics model were proposed by Oser (1991) in Tirri (1999). They were initially used as a template for reviewing teachers’ ethics. Three types of ethics were reported according to Oser (1991): normative, situational, and professional. Non-ethical, functional, and professional actions are all linked to professional ethics. Teachers’ ethical issues were recognized by Rique and Lins-Dyer (2003), whereas Ye and Law (2019) used a model of three types of moral claims (justice, caring, and truthfulness) for teachers to handle ethical dilemmas.

Further, Thornberg (2008) stated the lack of expertise in value education, exploring preventive or proactive approaches, designing and implementing clear rules or standards of behavior, creating a sense of community among students, and creating a positive school-wide climate and primary response methods for teachers.

Tirri (1999) investigated the strategies used by teachers to overcome ethical dilemmas using a quantitative mental questionnaire and reported that according to the characteristics of the moral challenge, teachers primarily used the following methods to resolve ethical conflicts: single-shot decisions, discursive decisions, delegated decisions, and avoiding making decisions. Teachers choose one of these strategies. Individual decisions were made when teachers wanted to resolve tensions authoritatively.

Soleimani and Lovat (2019) argued that understanding ethics could prepare one for better performance and better fulfillment of responsibilities. Based on teachers’ perceptions and strategies when facing ethical challenges in schools, Soleimani and Lovat (2019) clarified the moral dimensions of teaching and learning and presented the principles in teachers’ argumentation, showing a particular interest in adopting what she described as the “Fielder invariant” and “Felder-dependent” categories of ethical argumentation. Discursive strategies were used in situations where teachers were criticized for uneven judgments.

Thus, Tirri (1999) proposed teachers’ perceptions and strategies when facing moral challenges in schools, which articulated a framework for the moral dimension of teaching and learning. It involved in-depth study of today’s teachers’ perceptions of the moral curriculum of other moral development theories, such as the difficulties faced by teachers in implementing the moral curriculum.

#### Teaching Strategy

**Figure 7:**
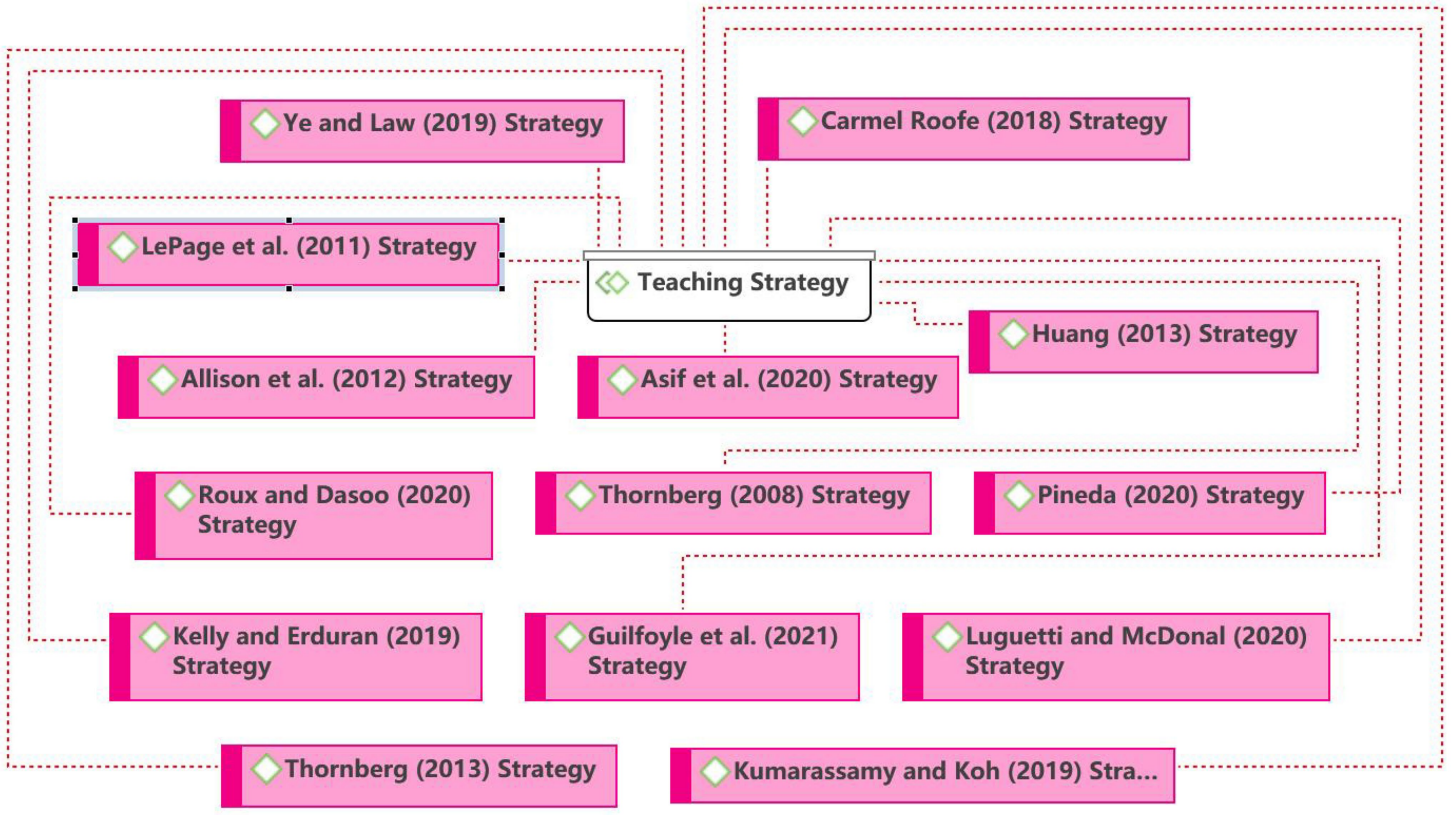
Network on the teaching strategy theme.

As seen in Figure 7, Ye and Law (2019) argued for teaching practice and the moral dilemma discussion approach to achieve pre-service teachers’ perception of moral education. Guilfoyle et al. (2021) argued that debate could be used as a strategy in secondary school teachers’ argumentative views on science and religious education. Thornberg (2008) stated the lack of expertise in value education and explored preventive or proactive approaches, designing and implementing clear rules or standards of behaviors, creating a sense of community among students, and creating a positive school-wide climate and primary response methods for teachers. Luguetti and McDonald (2020) used radical pedagogical approaches to explore pre-service teachers’ perceptions of love for young people from socially disadvantaged backgrounds. Teaching strategies or approaches have always been a critical area of research in teaching and learning.

Teachers must be prepared, whether they use a standard teaching technique or a progressive, constructivist, or critical teaching approach. According to Thornberg et al. (2013), the conventional path focuses on adults’ transmission of social morality through character education, direct instruction, exhortation, incentives, and punishments. Through processes of social interaction and moral discourse, progressive or constructivist methods “stress children’s active creation of moral meaning and development of a personal commitment to principles of justice and care for the welfare of others” (Solomon et al., 2001). According to a critical perspective, moral influences in schools, particularly in terms of school discipline and concealed curriculum, can be questioned and have far-reaching impacts without being detected (Giroux and Penna, 1979; Jones, 2009). As a result, instructors must be conversant with the advantages and drawbacks of conventional, progressive/constructivist, and critical approaches to values teaching (Thornberg et al., 2013).

Further, Thornberg (2008) stated the lack of expertise in value education, exploration of preventive or proactive approaches, design and implementation of clear rules or standards of behavior, creation of a sense of community among students, and creation of a positive school-wide climate and primary response methods for teachers.

Moreover, appropriate teaching strategies are to be adopted in different disciplines. Kelly and Erduran (2019) argued that using inquiry-based teaching methods, argumentation, and examples from the history of science in teaching can also describe a scenario. Roux and Dasoo (2020) stated that the element of play be used as a strategy for education. Moreover, it is possible to teach by modeling values (learning by example). Hung (2013) stated the use of the negotiated democracy model to teach moral education courses. LePage et al. (2011) and Asif et al. (2020) used problem-based approaches, the Socratic method, drama and service learning, and case studies to teach moral education. Students develop problem-solving and critical thinking skills.

Further, Kumarassamy and Koh (2019) stated the use of a student-centered approach. Allison et al. (2012) and Roofe (2018) used an interdisciplinary approach and research on relevant issues to teach. Pineda and Meier (2020) noted that action research and moral dilemmas can be conducted using open-ended questions. It is conducive to solving the problems posed by ethical dilemmas.

Therefore, teaching strategies are essential for moral education courses or other courses. There are many factors for teachers to consider in using teaching strategies. It seems urgent that they should try to find appropriate teaching strategies for students to learn and for the development in the process, especially in moral education courses. For example, narratives have become increasingly popular as an education teaching strategy in recent years, and researchers can focus on this aspect of research (Saharuddin et al., 2020, 2021).

#### The Role of the Teacher

**Figure 8:**
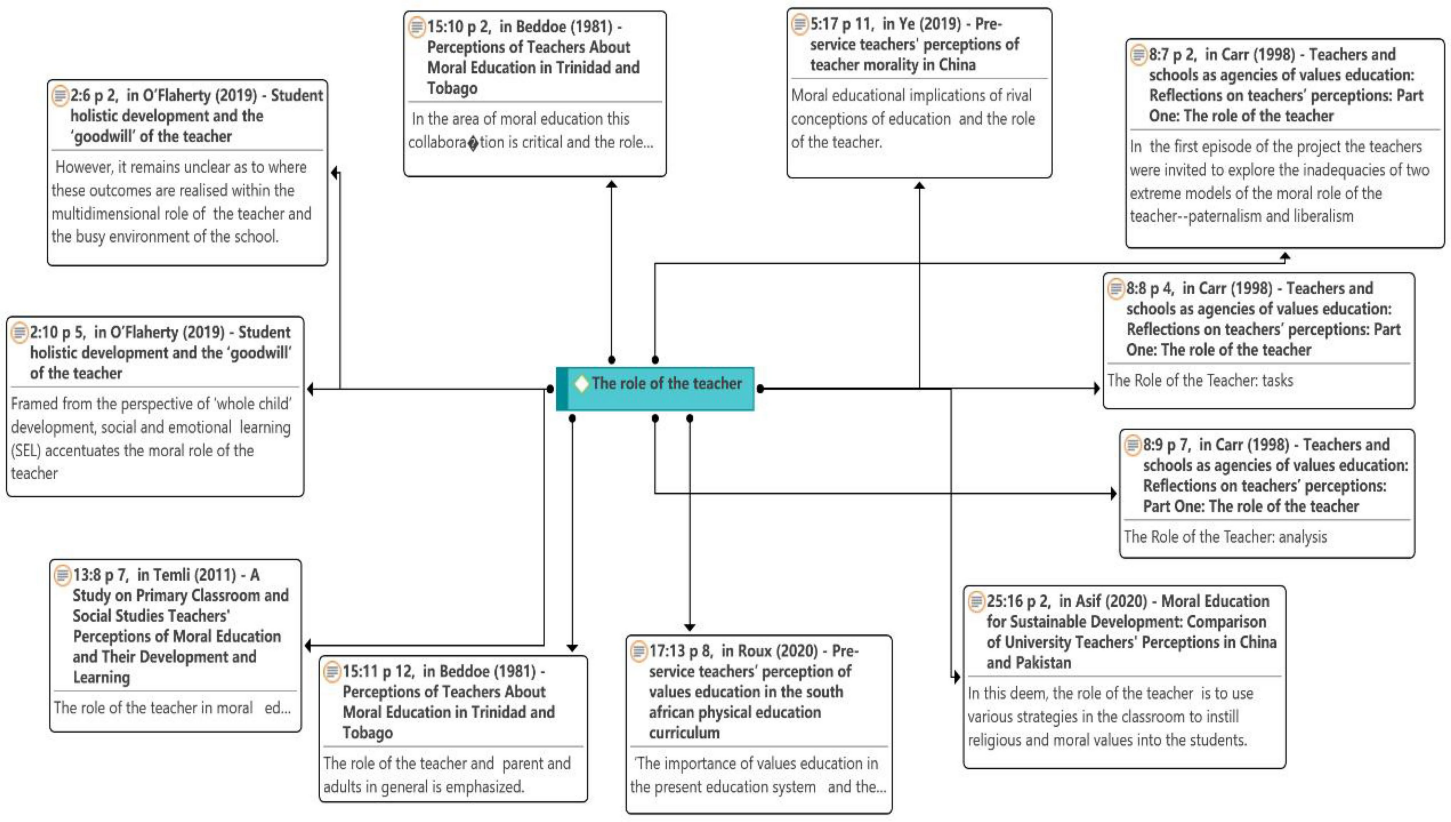
Network on the role of the teacher theme.

As seen in Figure 8, O’Flaherty and McCormack (2019) noted that it remains unclear where these outcomes will be achieved in the multidimensional role of teachers and busy school environment. In the study of Carr and Landon (1998, 1999) on the role of the teacher, teachers were invited to explore the inadequacies of two extreme models of the moral role of the teacher: paternalism and liberalism. This is where the teaching comes into play: task and analysis. The apparent conceptual link between moral education questions leadership and freedom, and it raises more profound moral epistemological questions about the objectivity or otherwise of moral judgments: whether these judgments can be perceived as correct or incorrect in principle. This has begun to emerge as a standout in the argument about the moral role of teachers.

However, the two approaches appear to be equally problematic for reasons that have nothing to do with earlier contractual or voluntary ethical considerations concerning instructors’ ethical duty. These techniques have the potential to result in genuine injustices, which are difficult to ignore. After debating the moral role of instructors, we decided that maintaining a true vision of human moral existence was more important than the lack of objectivity in reaching an accord. For the same reason, it is arguably important to develop a suitable understanding of the ethical duty of the teacher that is founded on a proper principled analysis of the objective reality of moral and other value judgments, rather than on popular opinion.

Ye and Law (2019) considered the impact of opposing educational concepts and role of the teacher on moral education. Temli et al. (2011) stated that teachers play an important role in moral education in primary classrooms and social studies of teachers’ conceptions of moral education and their development and learning. Beddoe (1981) suggested that in moral education, this collaboration is crucial, and the role of the teacher is vital. In general, the roles of teachers, parents, and adults are emphasized. Roux and Dasoo (2020) suggested the importance of value education in the current educational system and impact of the role of teachers on value education. Asif et al. (2020) indicated that the role of teachers is to use various strategies in the classroom to instill religious and moral values in students.

Teachers use instructional strategies, and there is no such thing as a good or bad instructional strategy. Teachers should find teaching methods that work for them and for their students’ development; however, they should focus on a combination of traditional teaching strategies, progressive/constructivist approaches, and critical teaching. Therefore, the teacher has a vital role in the moral education curriculum. It directly affects the quality and effectiveness of teaching and learning and development of the teachers themselves.

## A Proposed Conceptual Framework for Teachers’ Perceptions of Moral Education Curriculum

**Figure 9:**
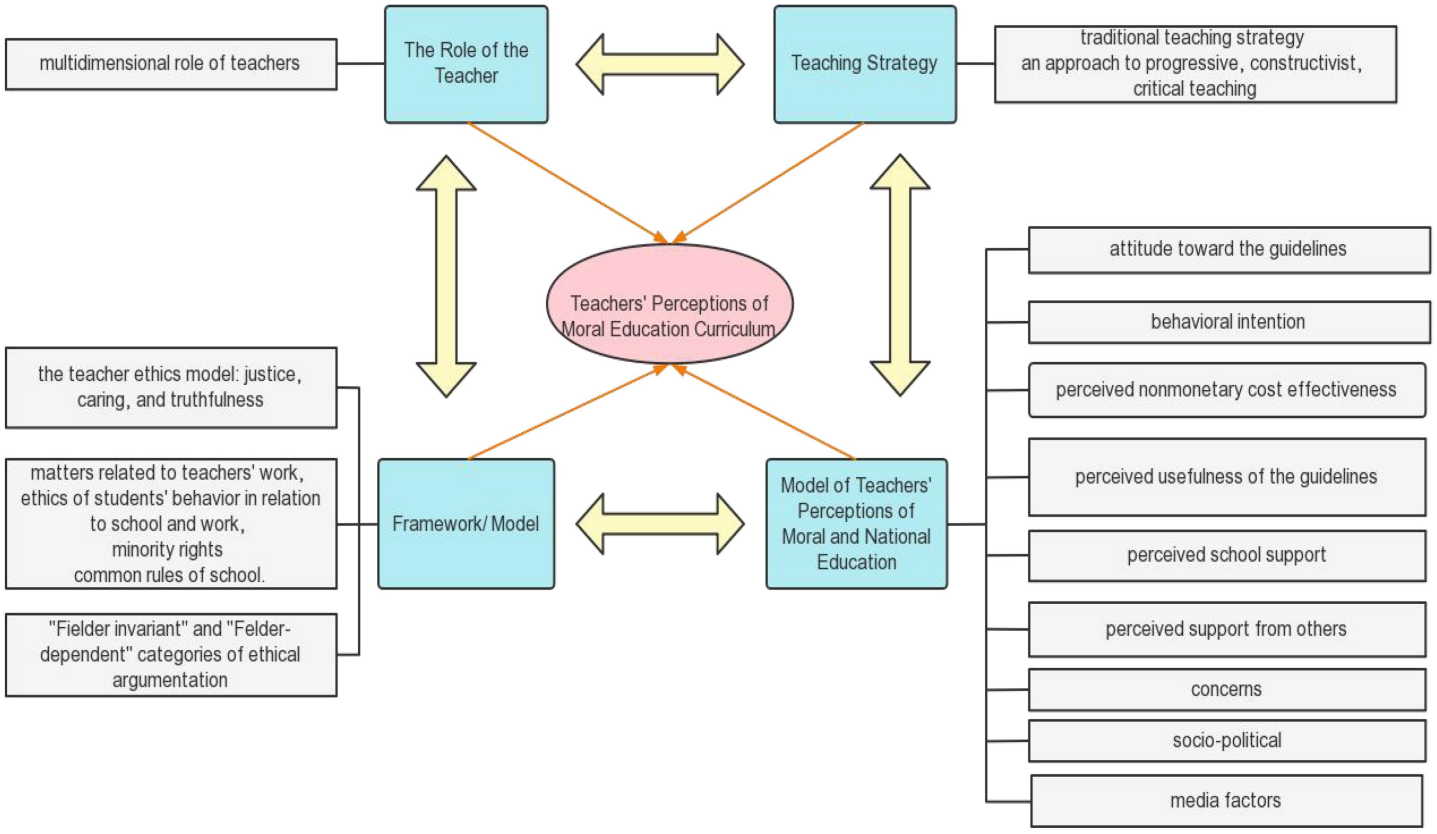
A conceptual framework for teachers’ perceptions of moral education curriculum.

Thematic reviews have resulted in new research proposals to continue developing data in this area. These propositions are indicated by reading, reviewing research, and definitions according to the conceptual framework shown in Figure 9. Figure 9 provides 32 research directions to guide teachers’ perceptions of moral education curriculum to discover new research opportunities, promote the development of moral education curriculum and teachers, facilitate policymakers to formulate relevant policies better, and encourage teachers’ awareness of moral education curriculum. A realistic approach is given to perception patterns. Given the current research scenario and planned structure, future research can be divided into:

1. *Model of teachers’ perceptions of moral and national education:* Variables or factors involving attitudes to guidelines, behavioral intentions, perceived non-monetary cost-benefits, perceived guideline usefulness, perceived school support, perceived others support and concerns, and socio-political and media factors. First, the environmental background should be a thoroughly understood, and the factors that affect teachers’ perception of moral education should be focused. These factors are optional, and elements can also be added.
2. *Frameworks and models*: A new framework combining theories of moral development with other ideas. Focus should be given on Oser (1991) model of teacher morality: justice, caring, and authenticity, along with Tirri (1999) matters related to teachers’ work, student conduct ethics in school and work, minority rights, standard rules in schools, and the “field invariant” and “field-dependent” categories of ethical arguments. Difficulties and challenges faced by teachers in implementing moral education should be dealt with.
3. *Teaching strategies*: It should focus on traditional teaching strategies, a progressive/constructivist approach, and a combination of critical teaching. Further, based on this, appropriate teaching methods can be explored for student development.
4. *The role of the teacher*: The multidimensional role of the teacher should be focused, and professional development of teachers should be learnt.

## Conclusion

This paper adopted two approaches based on the 32 articles reviewed in this paper. The first is the quantitative section, highlighting data obtained numerically from ATLAS.ti 9. Despite the growing interest in the topic, no review papers are available combining teachers’ perception with the literature on moral education curricula. Moreover, teachers’ perceptions and concepts should be adapted in the moral education curriculum literature to design and apply more systematic terminology. The research progress is still relatively slow, which is why the attention is lacking to the topic and status of the moral education curriculum. Additionally, teachers are affected and restricted by various factors, and their professionalism is notably lacking. In the qualitative part, the theme emphasizes the need to clarify the relationship between teachers’ perceptions and the moral education curriculum literature and find the factors that affect teachers’ perceptions in practice by studying teachers’ perceptions of the moral education curriculum. Teachers’ perceptions of moral education courses should be predicted and their professional and moral education development should be improved. Several publications suggest using teacher perception models of moral education programs to support research methods and frameworks to clarify the implementation process in actual practice.

The main contribution of this paper is that it examined the literature on teachers’ perception of moral education curriculum. The significant contribution of this paper was that it suggested improvements and introduced a new combination mode, explored the factors that affect teachers’ perception of moral education curriculum, and proposed a new research method or idea based on teachers’ perception of moral education curriculum. To make research more flexible, attention needs to be paid to the role of teachers and use of teaching strategies when examining teachers’ perceptions of moral education programs. This paper focused on patterns, frameworks, or patterns of teachers’ perception of the moral education curriculum; teachers’ roles and teaching strategies; and their existing critical practices in the moral education curriculum. Therefore, it is necessary to study and propose new models based on the existing ones. However, it must explain the relationship between teachers’ perceptions and moral education curriculum to reduce the bad influence on the implementation of moral education curriculum or solve difficulties and challenges by analyzing teachers’ perceptions. However, in this study, we only reviewed relevant literature and conducted a thematic analysis to obtain a framework for teachers’ perceptions of the moral education curriculum. The framework has not been specifically implemented and tested. In future studies, researchers should try to use the framework to study issues related to teachers’ perceptions of the moral education curriculum. Future research may continue to focus on teachers’ perceptions of moral education curriculum, develop and innovate models, and form theoretical systems.

## Data Availability Statement

The original contributions presented in the study are included in the article/supplementary material, further inquiries can be directed to the corresponding author.

## Author Contributions

QZ read relevant literature, organized and analyzed literature, and wrote the paper. NS and NA provided insightful suggestions and revisions to the manuscript. All authors contributed to the article and approved the submitted version.

## Conflict of Interest

The authors declare that the research was conducted in the absence of any commercial or financial relationships that could be construed as a potential conflict of interest.

## Publisher’s Note

All claims expressed in this article are solely those of the authors and do not necessarily represent those of their affiliated organizations, or those of the publisher, the editors and the reviewers. Any product that may be evaluated in this article, or claim that may be made by its manufacturer, is not guaranteed or endorsed by the publisher.
